# The Antioxidant Activity of New Coumarin Derivatives

**DOI:** 10.3390/ijms12095747

**Published:** 2011-09-07

**Authors:** Abdul Amir H. Kadhum, Ahmed A. Al-Amiery, Ahmed Y. Musa, Abu Bakar Mohamad

**Affiliations:** 1 Department of Chemical and Process Engineering, Faculty of Engineering and Built Environment, Universiti Kebangsaan Malaysia, Bangi, Selangor 43600, Malaysia; E-Mails: amir@eng.ukm.my (A.A.H.K.); ahmedym@eng.ukm.my (A.Y.M.); drab@eng.ukm.my(A.B.M.); 2 Biotechnology Division, Applied Science Department, University of Technology, Baghdad 10066, Iraq

**Keywords:** antioxidant, ethyl bromoacetate, 4-hydroxycoumarin, maleic anhydride

## Abstract

The antioxidant activity of two synthesized coumarins namely, *N*-(4,7-dioxo-2- phenyl-1,3-oxazepin-3(2*H*,4*H*,7*H*)-yl)-2-(2-oxo-2*H*-chromen-4-yloxy)acetamide **5** and *N*-(4-oxo-2-phenylthiazolidin-3-yl)-2-(2-oxo-2*H*-chromen-4-yloxy)acetamide **6** were studied with the DPPH, hydrogen peroxide and nitric oxide radical methods and compared with the known antioxidant ascorbic acid. Compounds **5** and **6** were synthesized in a good yield from the addition reaction of maleic anhydride or mercaptoacetic acid to compound **4,** namely *N′*-benzylidene-2-(2-oxo-2*H*-chromen-4-yloxy)acetohydrazide. Compound **4** was synthesized by the condensation of compound **3**, namely 2-(2-oxo-2*H*-chromen-4-yloxy) acetohydrazide, with benzaldehyde. Compound **3,** however, was synthesized from the addition of hydrazine to compound **2,** namely ethyl 2-(2-oxo-2*H*-chromen-4-yloxy)acetate, which was synthesized from the reaction of ethyl bromoacetate with 4-hydroxycoumarin **1**. Structures for the synthesized coumarins **2**–**6** are proposed on the basis of spectroscopic evidence.

## 1. Introduction

Coumarin and its derivatives represent one of the most active classes of compound possessing a wide spectrum of biological activity [1–4]. Many of these compounds have proven to be active as antibacterial [5–7], antifungal [8], anti-inflammatory [9], anticoagulant [10], anti-HIV [11] and antitumor agents [12]. Coumarins are widely used as additives in food, perfumes, cosmetics [13], pharmaceuticals and optical brighteners [14] and would dispersed fluorescent and laser dyes [15]. Coumarins also have the super thermal stability and outstanding optical properties including extended spectral response, high quantum yields and superior photo stability. Optical applications of these compounds, such as laser dyes, nonlinear optical chromophores, fluorescent whiteners, fluorescent probes, polymer science, optical recording and solar energy collectors have been widely investigated [16–20]. Classical routes to coumarins incorporate Pechmann, Knoevenagel, Perkin, Reformatsky, and Wittig condensation reactions [21–24]. To make these classical reactions efficacious, several variations in terms of catalyst and reaction conditions have been introduced [25,26]. 4-Hydroxycoumarin and its derivatives have been effectively used as anticoagulants for the treatment of disorders in which there is excessive or undesirable cloterting, such as thrombophlebitis [27], pulmonary embolism [28], and certain cardiac conditions [29]. Several comparative pharmacological investigations of the 4-hydroxycoumarin derivatives have shown it to have good anticoagulant activity combined with low side effects and little toxicity [30]. Antioxidants possess the ability to protect the cellular organelles from damage caused by free radicals induced oxidative stress. Free radicals used include hydroxyl radical, superoxide anion radical and hydrogen peroxide. Highly reactive free radicals which are formed by exogenous chemicals, stress or in the food system are capable of oxidizing biomolecules, resulting in cancer, coronary heart disease and hypertension [31]. Generally, most of the free radicals generated from metabolism are scavenged by endogenous defense system such as catalase, superoxide dismutase and peroxidase–glutathione system [32].

The preparation of ethyl 2-(2-oxo-2*H*-chromen-4-yloxy)acetate **2**, which was used as a starting material for the synthesis of compounds **5** and **6**, is presented in this study. The chemical structures of the synthesized compounds **2**–**6** were conducted and confirmed. The *in vitro* antioxidant activities were investigated for the synthesized compounds **5** and **6**.

## 2. Results and Discussion

### 2.1. Chemistry

**Scheme 1:**
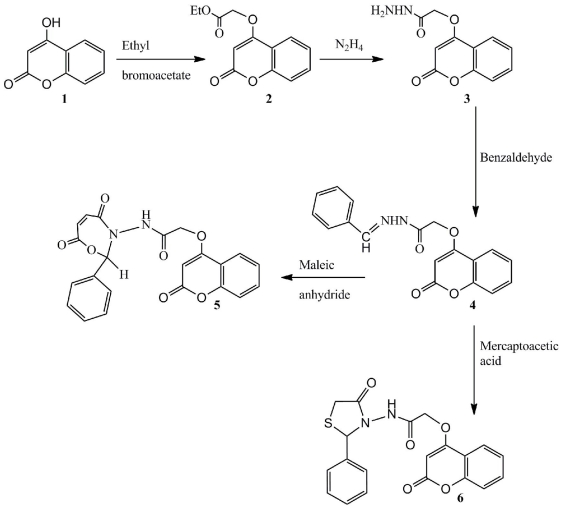
Reaction sequences of compounds **5** and **6**.

The reaction sequence for the synthesis of *N*-(4,7-dioxo-2-phenyl-1,3-oxazepin-3(2*H*,4*H*,7*H*)-yl)-2- (2-oxo-2*H*-chromen-4-yloxy)acetamide **5** and *N*-(4-oxo-2-phenylthiazolidin-3-yl)-2-(2-oxo-2*H*chromen-4-yloxy)acetamide **6**, is out lined in Scheme 1.

The synthesis of ethyl 2-(2-oxo-2*H*-chromen-4-yloxy)acetate **2** was conducted via reflux of Ethyl bromoacetate with 4-hydroxycoumarin **1** in the presence of anhydrous potassium carbonate in acetone. Hydrazinolysis of compound **2** with hydrazine hydrate produced hydrazide **3** in good yield. The FT-IR spectrum of compound **3** showed absorption bands in the 3297.3, 3211 cm^−1^ (hydrazide NH-NH_2_), 1711 cm^−1^ (lactonic -C=O carbonyl stretching), and 1671.2 cm^−1^ (amide -C=O carbonyl stretching). The ^1^H-NMR spectrum exhibited a singlet due to the -CO-NH-NH_2_ proton at δ 8.89 ppm. Compound **3** was thencondensed with benzaldehyde to make compound **4** that can be cyclized with maleic anhydride (or mercaptoacetic acid)to yield compounds **5** (and **6**). The IR spectrum of compound **4** showed characteristic bands at 3191.1 cm^−1^ (NH), 1734.3 (C=O, lactone) cm^−1^, 1688 cm^−1^ (C=O, amide). The ^1^H-NMR spectrum showed the absence of NH_2_ protons, and the presence of the N=CH proton at 8.23 ppm. Compound **5** shows pronounced peaks; a singlet due to the NH proton at 9.58 in addition to a singlet for 1H assigned to the (-C=C-H). The IR spectrum of compound **6** showed characteristic bands at 3198.7 cm^−1^, 29211731 cm^−1^, and 1689 and 1667 cm^−1^ (NH), (C-H alkane), (C=O, lactone) and (C=O, amide) respectively. The ^1^H-NMR spectrum of compound **6** showed peaks at 5.87 ppm (s, 1H) =C-H and 3.91 (d, 2H, CH_2_).

**Scheme 2:**
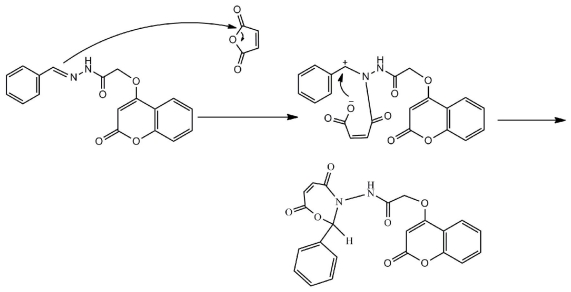
Mechanism of formation of compound 5.

The reaction of compound **4** with maleic anhydridecould be visualized through the mechanism shown in Scheme 2.

#### 2.1.1. Computational Studies

**Figure 1:**
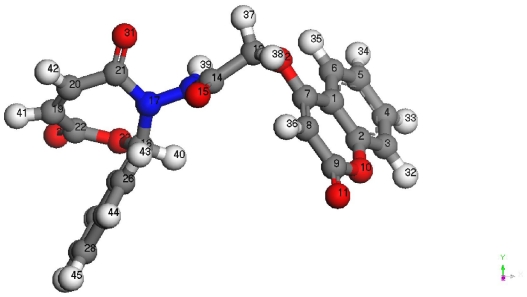
Optimized 3D geometrical structure for compound **5**.

An earlier study [33] has shown that atomic charges were affected by the presence of the substituent of rings. With the aid of a reference model, compound anoptimized geometry and the 3D geometrical structureis given in Figure 1 and Table 1, respectively. The data obtained show that the heat of formation are about 81.154956 kJ/mol) and the highest atomic charge in compound **5** is at [O(12) −0.510] followed by the next charge value at [O(10) −0.509] and [O(15) (−0.437)]. These data show clearly that these three atoms are the most reactive toward the addition, substitution reactions and bonding with the metal. The determined bond angle and twist angle, stretch (1.4560), bend (4.3225), stretch-bend (−0.1213). The 3D geometrical structure indicates that this molecule is a non-planarmolecular and the stereochemistry is [C(18): (S); C(19)–C(20): (E); C(7)–C(8): (Z)].

### 2.2. Pharmacology

#### 2.2.1. Antioxidant Activity

**Figure 2:**
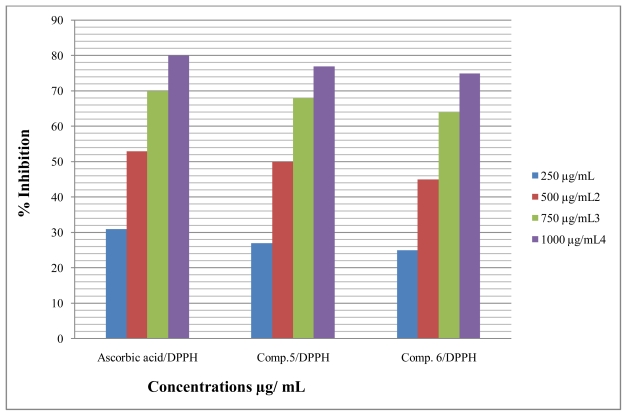
Effect of compound **5** and **6** toward 1,1-diphenyl-2-picrilhydrazyl (DPPH).

**Figure 3:**
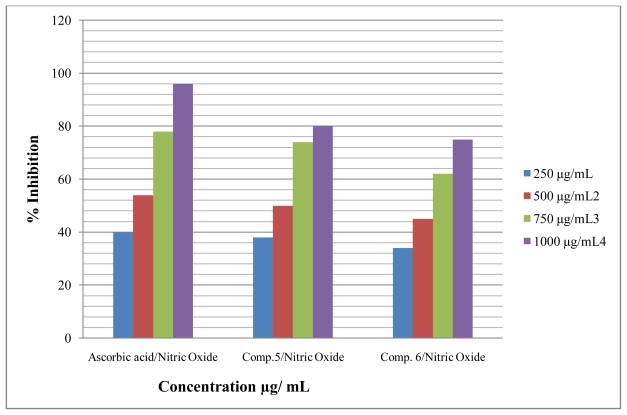
Effect of compound **5** and **6** toward nitric oxide.

**Figure 4:**
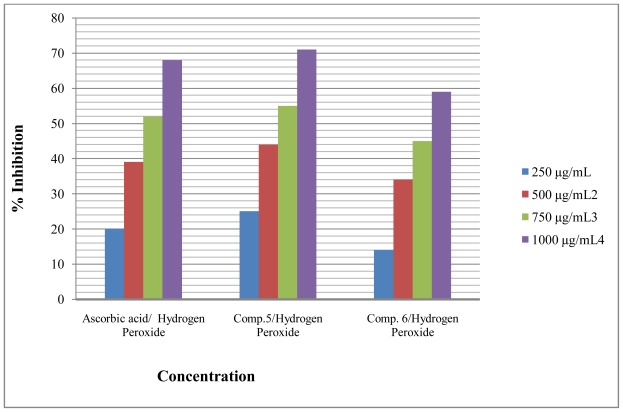
Effect of compound **5** and **6** toward hydrogen peroxide.

Antioxidant compounds in food play an important role as a health-protecting factor. Scientific evidence suggests that antioxidants reduce the risk for chronic diseases including cancer and heart disease. Primary sources of naturally occurring antioxidants are whole grains, fruits and vegetables. Plant-sourced food antioxidants like vitamin C, vitamin E, carotenes, phenolic acids, phytate and phytoestrogens have been recognized as having the potential to reduce disease risk. Most of the antioxidant compounds in a typical diet are derived from plant sources and belong to various classes of compounds with a wide variety of physical and chemical properties. Some compounds, such as gallates, have strong antioxidant activity, while others, such as the mono-phenols are weak antioxidants [34]. The role of antioxidant is to remove free radicals. One important mechanism through which this is achieved is by donating hydrogen to free radicals in its reduction to non-reactive species. Addition of hydrogen would remove the odd electron feature which is responsible for radical reactivity. Free radicals have been a subject of significant interest among scientists in the past decade. Their broad range of effects in biological systems has drawn the attention of many workers. It has been proven that free radicals play an important role in the pathogenesis of certain diseases and aging. There are many reports that support the use of antioxidant supplementation in reducing the level of oxidative stress and in slowing or preventing the development of complications associated with diseases [35]. Many synthetic antioxidant components have shown toxic and/or mutagenic effects, and there-fore attention has been paid to naturally occurring antioxidants. Compound **5** and **6** were screened for *in vitro* antioxidant activity using (1,1-diphenyl-2-picrilhydrazyl) DPPH, Nitric oxide and hydrogen peroxide. They show good antioxidant activity against all methods (Figures 2–4). The hydrogen-donating activity, measured using DPPH radicals as hydrogen acceptor, showed that significant association could be found between the concentration of the novel molecule and the percentage of inhibition. Through DPPH test, compounds **5** and **6** have down to reduce the stable radical DPPH to the yellow-colored diphenylpicrylhydrazine. The method was based on the reduction of alcoholic DPPH solution in the presence of a hydrogen-donating antioxidant due to the formation of the non-radical form DPPH-H in the reaction [36]. The nitric oxide assay has been widely used to evaluate the effectiveness of the free radical scavenging on various antioxidant substances. Nitric oxide generated as a result of decomposition of sodium nitroprusside in aqueous medium interacts with oxygen at physiological pH to produce nitrite ions. The nitrite ions were subjected to diazotization followed by azo coupling reaction to yield an azo dye measured by an absorption band at 540 nm. The scavenging ability of the synthesized compounds **5** and **6** was compared with ascorbic acid as a standard. Nitric oxides radical inhibition study showed that the synthesized compounds were a potent scavenger of nitric oxide. The compounds **5** and **6** inhibited nitrite formation by competing with oxygen to react directly with nitric oxide and also to inhibit its synthesis. Scavengers of nitric oxide competed with oxygen, leading to the reduced production of nitric oxide [37].

**Scheme 3:**
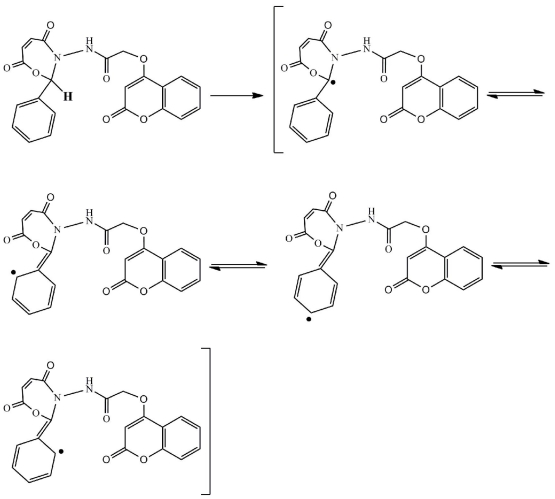
Suggested mechanism for compound **5** as antioxidant.

**Scheme 4:**
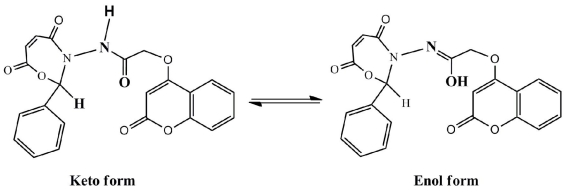
Suggested mechanism for compound **5** fellow the route of the keto-enol forms.

There are two postulated mechanisms for the reaction of compound **5** as an antioxidant as shown in Schemes 3 and 4. The first mechanism depends on the benzyl hydrogen atom (bold hydrogen atom), where this atom was under the influence of two effects, namely resonance and inductive. The resonance effect of benzyl hydrogen makes the release of hydrogen as a free radical easy while the inductive effect on benzene ring, oxygen and nitrogen pushes the electrons toward a carbon free radical, resulting in the molecule becoming stable.

The second postulated mechanism fellows the route of the keto-enol forms as shown in Scheme 4.

**Scheme 5:**
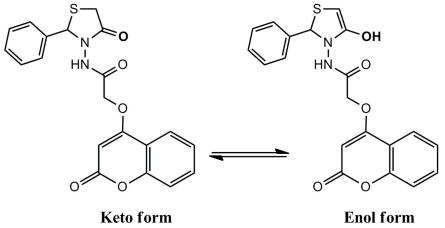
Suggested mechanism for compound **6** fellow the route of the keto-enol forms.

**Scheme 6:**
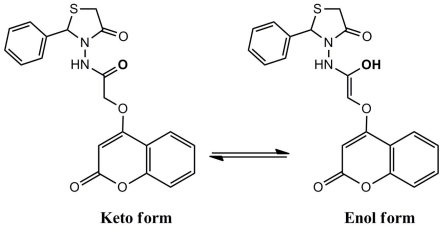
Suggested mechanism for compound **6** fellow the route of the keto-enol forms

For compound **6**, the two suggested mechanisms depend on the keto-enol form as depicted on Schemes 5 and 6.

## 3. Experimental Section

### 3.1. General

All chemical**s** used were of reagent grade (supplied by either Merck or Fluka) and used as supplied without further purifications. The FTIR spectra were recorded as KBr disc on FTIR 8300 Shimadzu Spectrophotometer. The UV-Visible spectra were measured using Shimadzu UV-VIS. 160A spectrophotometer. Proton NMR spectra were recorded on Bruker - DPX 300 MHz spectrometer with TMS as the internal standard. Elemental micro analysis was carried out using a CHN elemental analyzer model 5500-Carlo Erba instrument.

### 3.2. Chemistry

#### 3.2.1. Synthesis of Ethyl 2-(2-oxo-2*H*-chromen-4-yloxy)acetate **2**

A suspension of 4-hydroxycoumarins **1** (6.17 mmol) in acetone (30 mL) was refluxed with ethyl bromoacetate (9.15 mmol) and K_2_CO_3_ (4.69 g, 33.91 mmol) for 12 h. After cooling, the mixture was evaporated to dryness and the residue was partitioned between CHCl_3_ (50 mL) and water (50 mL). The organic phase was dried (Na_2_SO_4_), filtered and evaporated to dryness. The residue was recrystallized from acetone; yield 92%; m.p. 99.0 °C; ^1^H-NMR (CDCl_3_): δ 3.22 (t, 3H, CH_3_), δ 3.85 (m, 2H, CH_2_), δ 4.91 (s, 2H) and δ 5.20, *δ* 5.250, δ 5.272 (s, 2H) for CH_2_), δ 5.78 (s, 1H) for -C=C-H), δ 7.291, δ 7.478, δ 7.80 (s, 1H) for aromatic ring); ^13^C-NMR: 167.2; 165.1; 163.4, 155.9; 134.2; 121.8; 121.1; 119.0; 113.8; 100.9; 65.3; 54.7; 22.12; IR: 2987.3 cm^−1^ (C-H, Aliphatic), 3089.5 cm^−1^ (C-H, Aromatic), 1759.3 cm^−1^ (C=O, Lactonic), 1717.6 cm^−1^ (C=O, Estric), 1629.2 cm^−1^ (C=C, Alkene), 1577.6 cm^−1^ (C=C, Aromatic); Theoretical Calculation for C_13_H_12_O_5_: C 62.90%, H 4.87%. Experimental: C 61.91% H 3.99%.

#### 3.2.2. Synthesis of 2-(2-oxo-2*H*-chromen-4-yloxy)acetohydrazide **3**

A solution of compound **2** (10 mmol) in ethanol 25 mL was refluxed with hydrazine hydrate (15 mmol) for 4 h. After concentrating the reaction mixture a solid mass separated out and recrystallized using ethanol, yield 51%, m.p. 228 °C; ^1^H-NMR (CDCl_3_): δ 4.48 (s, 2H, CH_2_), δ 4.65 (s, 2H, NH_2_), δ 8.89 (s, 1H, NH), δ 4.92 (s, 2H) and *δ* 5.210 (s, 2H) for (O-CH_2_), δ 5.72 (s, 1H) for (-C=C-H), δ 7.410, δ 7.521, δ 8.10 (s, 1H) for aromatic ring; IR: 3297.3, 3211 cm^−1^ (N-H), 2906.0 cm^−1^ (C-H, Aliphatic), 3072.7 cm^−1^ (C-H, Aromatic), 1711.5 cm^−1^ (C=O, Lacton), 1671.2 cm^−1^ (C=O, Amide); Theoretical Calculation for C_11_H_10_N_2_O_4_: C 56.41%, H 4.30%, N 11.96%. Experimental: C 57.13% H 4.01%, N 10.52%.

#### 3.2.3. Synthesis of *N*′-benzylidene-2-(2-oxo-2*H*-chromen-4-yloxy)acetohydrazide **4**

A solution of the compound **3** (0.2 mmol) in ethanol 25 mL was refluxed with benzaldehyde (0.2 mmol) for 20 h. After cooling to room temperature, a solid mass separated and recrystallized. Recrystallized from ethanol;yield 57%; m.p. 258 °C; ^1^H-NMR (CDCl_3_): δ 8.23, (s, 1H,N=CH), δ 8.11 (s, 1H, NH), δ 4.91 (s, 2H) and δ 5.340 (s, 2H) for O-CH_2_), δ 5.67 (s, 1H) for (-C=C-H), δ 7.240, δ 7.312, δ 7.97 (s, 1H) for aromatic ring; ^13^C-NMR (DMSO-d6): 170.0, 169.2, 164.1, 155.1,145.6, 136.9,129,8, 128, 125, 120.6, 119.9, 118.4,118.3, 117.9, 80.1, 65.2; IR: 3191.1cm^−1^ (N-H), 2901.0 cm^−1^ (C-H, Aliphatic), 3058.2 cm^−1^ (C-H, Aromatic), 1743.3 cm^−1^ (C=O, Lacton), 1688 cm^−1^ (C=O, Amide), 1613 cm^−1^ (C=N), 1627.7 cm^−1^ (C=C); Theoretical Calculation for C_18_H_14_N_2_O_4_: C 67.07%, H 4.38%, N 8.69%. Experimental: C 66.39% H 3.95%, N 7.97%.

#### 3.2.4. Synthesis of *N*-(4,7-dioxo-2-phenyl-1,3-oxazepin-3(2*H*,4*H*,7*H*)-yl)-2-(2-oxo-2*H*-chromen-4-yloxy) acetamide **5**

Mixture of 1 mmole of compound **4** with 1 mmole of maleic anhydride in 50 mL of dry benzene was refluxed in water bath for 20 h. The solvent was removed and the precipitate was recrystallized from tetrahydrofuran; yield 44%; m.p. 201 °C; ^1^H-NMR (CDCl_3_): δ 3.93 and 4.22 (dd, 2H, CH_2_), δ 9.58 (s, 1H, NH), δ 4.81 (s, 2H) and δ 5.350 (s, 2H) for O-CH_2_), δ 6.11 (s, 1H) for (-C=C-H), δ 7.320, δ 7.412, δ 7.79 (s, 1H) for aromatic ring; ^13^C-NMR (DMSO-d6): 170.4, 167.2, 165.8, 160.6, 155.1, 155.4, 139.4, 136, 128.8, 125.8, 124.3, 120.6, 119.6, 117.1, 116.3 115.3, 113,100.6, 69.9, 55.4; IR: 3232.1cm^−1^ (N-H), 2979.5 cm^−1^ (C-H, Aliphatic), 3097.3 cm^−1^ (C-H, Aromatic), 1765, 1741 cm^−1^ (C=O), 1671 cm^−1^ (C=O, amide), 1617.8 cm^−1^ (C=N), 1632.6 cm^−1^ (C=C, Aromatic); Theoretical Calculation for C_22_H_16_N_2_O_7_: C 62.86%, H 3.84%, N 6.66%. Experimental: C 61.99% H 3.72%, N 5.90%.

#### 3.2.5. Synthesis of *N*-(4-oxo-2-phenylthiazolidin-3-yl)-2-(2-oxo-2H-chromen-4-yloxy)acetamide **6**

Mixture of 0.01 mole of Schiff base with 0.01 of mercaptoacetic acid in 50 mL of dry benzene was refluxed in water bath for 20 hours., filtered off, washed with water, dried and recrystallized from dichloromethane; yield 70%; m.p. 261 °C; ^1^H-NMR (CDCl_3_): δ 3.83 (, 2H, CH_2_), δ 5.40 (s, S-CH), δ 8.98 (s, 1H, NH), δ 4.77 (s, 2H) and δ 5.110 (s, 2H) for O-CH_2_), δ 5.82 (s, 1H) for (−C=C-H), δ 7.320, δ 7.441, δ 7.92 (s, 1H) for aromatic ring; ^13^C-NMR (DMSO-d6): 171, 169.0, 167.8, 154.1, 159.8, 128.2, 127.8, 126.1, 124.3, 120.1, 117.1, 114.4, 112.9,110.1 101.4, 77.6, 65.4, 33.2; IR: 3198.7cm^−1^ (N-H), 2921 cm^−1^ (C-H, Aliphatic), 3087.7 cm^−1^ (C-H, Aromatic), 1731 cm^−1^ (C=O, Lacton), 1689, 1667 cm^−1^ (C=O, amide), 1622 cm^−1^ (C=N), 1632.8 cm^−1^ (C=C, Aromatic); Theoretical Calculation for C_20_H_16_N_2_O_5_S: C 60.60%, H 4.07%, N 7.07%. Experimental: C 60.41% H 3.91%, N 6.88%.

### 3.3. Pharmacology

#### 3.3.1. (2,2-diphenyl-1-picrylhydrazyl) (DPPH) Radical Scavenging Activity

The DPPH radical scavenging activities of the test samples compounds (**5** and **6**) were evaluated according to Soares, *et al* [38]. Initially, 0.1 mL of the samples at concentration of 250, 500, 750 and 1000 μg/mL was mixed with 1 mL of 0.2 mM DPPH that was dissolved in methanol. The reaction mixture was incubated in the dark for 20 min at 28 °C. The control contained all reagents without the sample while methanol was used as blank. The DPPH radical scavenging activity was determined by measuring the absorbance at 517 nm using the UV-VIS spectrophotometer. The DPPH radical scavenging activity of ascorbic acid was also assayed for comparison.

The percentage of DPPH radical scavenger was calculated using Equation 1.

(1) $$Scanvenging\;effect(\%)=\frac{A_{0}-A_{1}}{A_{0}}\times 100$$

where *A*_0_ is the absorbance of the control reaction and *A*_1_ is the absorbance in the presence of the samples or standards.

#### 3.3.2. Nitric Oxide Scavenging Activity

Sodium nitroprusside in aqueous solution at physiological pH generates nitric oxide spontaneously; it interacts with oxygen to produce nitrite ions, which can be estimated by the use of GriessIllosvoy reaction [39]. In the present investigation, GriessIllosvoy reagent was modified using naphthylethylenediaminedihydrochloride (0.1% w/v) instead of 1-naphthylamine (5%). The reaction mixture (3 mL) containing sodium nitroprusside (10 mM, 2 mL), phosphate buffer saline (0.5 mL) and compounds **5** and **6** (250, 500, 750 and 1000 μg/mL) or standard solution (0.5 mL) was incubated at 25 °C for 150 min. After the incubation, 0.5 mL of the reaction mixture containing nitrite was pipetted and mixed with 1 mL of sulphanilic acid reagent (0.33% in 20% glacial acetic acid) and allowed to stand for 5 min for completing diazotization. Then, 1 mL of naphthylethylenediaminedihydrochloride (1%) was added, mixed and allowed to stand for 30 min. A pink-colored chromophore was formed in diffused light. The absorbance of these solutions was measured at 540 nm against the corresponding blank. Ascorbic acid was used as standard. Nitric oxide percentage scavenging activity was then calculated using Equation 1.

#### 3.3.3. Hydrogen Peroxide Scavenging Activity

A solution of hydrogen peroxide (40 mM) was prepared in phosphate buffer (pH 7.4). Different concentrations ((250, 500, 750 and 1000 μg/mL)) of synthesized compounds (or ascorbic acid) were added to a hydrogen peroxide solution (0.6 mL, 40 mM). Absorbance of hydrogen peroxide at 230 nm was determined after 10 min. against a blank solution containing phosphate buffer without hydrogen peroxide [40,41]. Hydrogen peroxide percentage scavenging activity was then calculated using Equation 1.

### 3.4. DFT

The molecular sketch of the reference compound was plotted using Visualization Materials Studio 5.5 software. All quantum chemical calculations were performed using the Density Functional Theory (DFT) in the methodology. DMol^3^ model was employed to obtain quantum chemical parameters and optimization of the molecule geometry. Molecular atomic charges were calculated by Mulliken population analysis.

## 4. Conclusions

In this study, the two coumarins, namely *N*-(4,7-dioxo-2-phenyl-1,3-oxazepin-3(2*H*,4*H*,7*H*)-yl)-2- (2-oxo-2H-chromen-4-yloxy)acetamide and *N*-(4-oxo-2-phenylthiazolidin-3-yl)-2-(2-oxo-2H-chromen-4-yloxy)acetamide, have been successively synthesized, and characterized by using various spectroscopic methods and elemental analysis techniques. Two postulated mechanisms have been proposed for the action of compound as antioxidant. The antioxidant activity of the compounds was initially tested and showed that the compounds have improved properties compared to ascorbic acid.

## Figures and Tables

**Table 1:** Atomic charge (Mulliken atomic charges) of synthesized compound **5**.

Atom	Charge	Atom	Charge	Atom	Charge	Atom	Charge
C (1)	0.044	C (13)	−0.138	C (25)	0.110	H (37)	0.189
C (2)	0.365	C (14	0.549	C (26)	−0.203	H (38)	0.188
C (3)	−0.196	O (15)	−0.437	C (27)	−0.112	H (39)	0.325
C (4)	−0.111	N (16)	−0.400	C (28)	−0.117	H (40)	0.182
C (5)	−0.125	N (17)	−0.264	C (29)	−0.112	H (41)	0.161
C (6)	−0.180	C (18)	0.185	C (30)	−0.180	H (42)	0.165
C (7)	413	C (19)	−0.160	O (31)	−0.465	H (43)	0.188
C (8)	−0.339	C (20)	−0.159	H (32)	0.147	H (44)	0.126
C (9)	0.556	C (21)	0.517	H (33)	0.130	H (45)	0.122
O (10)	−0.509	C (22)	0.560	H (34)	0.126	H (46)	0.123
O (11)	−0.426	O (23)	−0.499	H (35)	0.147	H (47)	0.152
O (12)	−0.510	O (24)	−0.411	H (36)	0.183	**-**	**-**
